# Does the Presence of Birdsongs Improve Perceived Levels of Mental Restoration from Park Use? Experiments on Parkways of Harbin Sun Island in China

**DOI:** 10.3390/ijerph17072271

**Published:** 2020-03-27

**Authors:** Xun Zhu, Ming Gao, Wei Zhao, Tianji Ge

**Affiliations:** 1 School of Architecture, Harbin Institute of Technology, Key Laboratory of Cold Region Urban and Rural Human Settlement Environment Science and Technology, Ministry of Industry and Information Technology, Harbin 150006, China; zhuxun@hit.edu.cn (X.Z.); 19s034123@stu.hit.edu.cn (M.G.); getj@vanke.com (T.G.); 2 China Vanke Co., Ltd., Vanke Center 33 Huanmei Road, Dameisha Yantian District, Shenzhen 518083, China

**Keywords:** urban park, birdsong, perceived restorative, path, soundscape

## Abstract

Green spaces in cities and urban parks serve as central areas for mental restoration and relieving pressure, and attention to soundscapes for their mental health benefits has become more prevalent. Birdsongs are perceived to enhance the restorative benefits of urban parks. This study examines Harbin Sun Island Park, the main bird habitat in the city of Harbin with numerous types of landscapes. We used space syntax to select the appropriate path space as a carrier and the pixel grid method to quantify path space shapes. A correlation analysis of field data was also used to explore the perceived restorative effects of birdsongs heard in urban parks using scales detailing the perceived restorative effects of various visual and auditory stimuli. The results show that soundscapes can significantly improve perceived recovery benefits, and that hearing birdsongs can significantly improve the perceived restorative benefits of wetland paths; the sky index of a tour path showed a significantly negative correlation with each feature (i.e., the four featured dimensions of “charm”, “escape”, “ductility” and “compatibility” included in the recovery scale), and the soft/hard ratio showed a significantly negative correlation with each studied feature. When the sky index ranged from 13–36%, tree coverage of the vertical coverage range was 30.28–38.6%, and when the soft/hard ratio ranged from 5–21, the perceived recovery benefit was strongest.

## 1. Introduction

Rapid urbanization has resulted in increased mental health problems among residents. Typical features of urban life such as excessive stimulation and information, tense and crowded spaces, and work and societal pressures are becoming more pronounced every day. Urban residents are in a constant state of mental tension and experience continual mental pressures. A number of studies such as Kaplan’s attention restoration theory [1] and Ulrich’s stress recovery theory [2] have confirmed the health effects of green spaces on residents. Green spaces have many proven benefits on physiology and psychology, which in turn have a significantly positive impact on human health by relaxing the body and mind and preventing mental fatigue (Kaplan, 1995). Compared to what is experienced in urban environments, people more easily recover from stress and attention fatigue in green spaces [3]. At the same time, green spaces can help alleviate stress and anxiety [4].

Urban green spaces are an essential element of public mental health and facilitate social interaction and public activities. Some European city managers believe that city parks provide residents with spaces for entertainment and interactive activities [5]. Residents of urban environments can more easily relieve pressure and attention fatigue when using natural spaces, and green spaces enhance residents’ levels of psychological adaptability [6], with the green coverage rate playing a role in the mental health of residents [7]. Jiang B et al. [8] found that the green vision rate with the strongest effect in terms of relieving stress ranges from 24% to 34%. Urban green space also plays a role in reducing noise and restoring mental wellbeing (e.g., attention span and mental health) [9] while encouraging healthy behaviors (e.g., sport and social activities) [10,11].

From these effects of green space, we develop and expand on related fields by studying the effects of birdsongs on resident health through audio-visual interactions based on the assumption that well-designed urban green spaces can promote the public health of urban residents [12,13]. The purpose of this study is to explore the perceived restorative effects of hearing birdsongs in green spaces. Taking the correlations between the recreational footpaths and path characteristics of Harbin Sun Island Park as an example, this study presents a comparative study of the perceived restorative effects achieved through audio-visual interaction, which was conducted to analyze the perceived restorative effects of birdsongs heard in urban parks.

At present, research on the effects of sound environment restoration shows that, compared to the artificial sounds of daily life activity, natural sounds are more comforting, and can evoke positive emotions [14]. Birdsongs and the sound of running water can make people feel especially happy and relieve psychological pressure, and constitute the main sounds heard in urban parks. In urban parks, natural sounds and scenes can promote physical and mental recovery in individuals experiencing stress and negative emotions, and specific sounds experienced in nature have recovery potential [15]. For this reason, researchers have evaluated the restorative effects of urban parks, and their results show that such effects are dependent on the scale of urban parks and on noise levels [16]. In recent years, scholars have conducted considerable research on soundscapes experienced in urban park green spaces [17,18]. There is a close relationship between perceptions and experiences with soundscapes in urban green spaces [19]. At the same time, visual landscapes may affect overall soundscape preferences through certain sounds [20].

As birdsongs are some of the most prominent animal sounds experienced in urban parks [21,22], research on the perceptual restoration of environmental soundscapes in parks has focused on birdsong soundscapes. Birdsongs have been studied in reference to their capacities to help relieve stress and restore attention [21,22,23]. In addition, some birdsongs are considered pleasant, while others are considered negative, such as the sound of seagulls. Such differences between birdsongs may lead to different perceived restorative effects.

Mynott and Cocker [24,25] noted that recovery benefits may be affected by cultural associations with certain birds, as individual species symbolize different features in different cultures. Jahnke [26] studied the recovery of attention after experiencing fatigue in office environments and found that those exposed to natural sounds (birdsongs) experience enhanced awareness and motivation to work than those exposed to sounds of an office environment. Through a semantic analysis, Yuan Zhang [27] found that the restorative effect of hearing birdsongs was the strongest of those in urban spaces, followed by the sound of running water and background music. Eleano [22,28] also made positive contributions to research on the effects of birdsongs regarding stress and perceived restorative effects. Birdsongs, as the sounds with the most restorative effects, had more restorative potential when experienced in urban green spaces [29].

This study considers whether watching green spaces and hearing birdsongs can effectively promote attention recovery and relieve stress.

## 2. Materials and Methods

**Figure 1:**
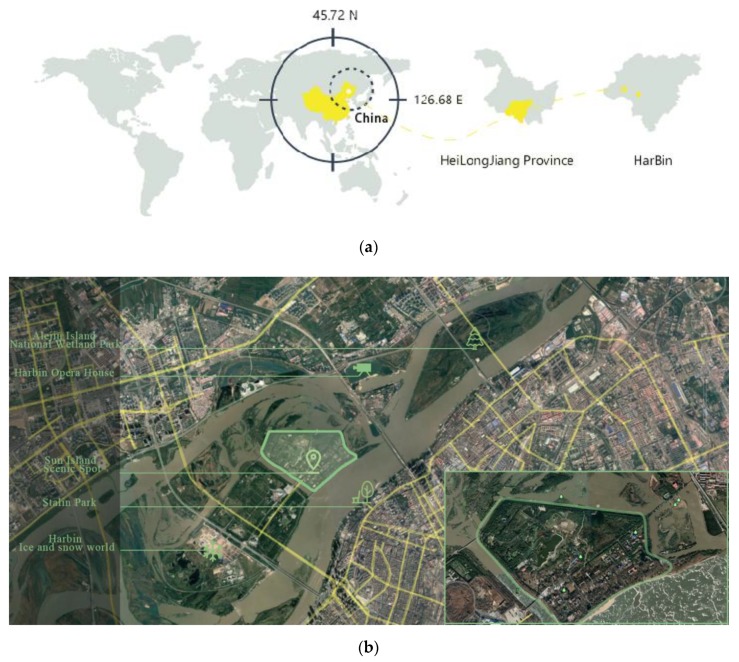
Site location analysis: (**a**) location analysis of Harbin; (**b**) geographical location of the experimental site.

The sample area used in this study is located in the core tourist area of the national 5A level scenic section of Sun Island of the city of Harbin, Heilongjiang Province, as shown in the site location analysis covering an area of 15 hectares (Figure 1). The island’s flat terrain forms part of a low flood plain. At high tide, Sun Island forms an archipelago; between high and low tide, it forms a complete island; and at low tide, it forms a peninsula. The site is rich in vegetation, includes a large water area, and has high levels of habitat heterogeneity. The environment provides habitat and migration channels for different birds. Data show that [30] 98 kinds of birds (including migratory birds) reside on the island. In view of research theories, we explore potential differences in perceived restorative effects of hearing birdsongs through audio-visual interactions.

### 2.1. Path Sample Selection

**Figure 2:**
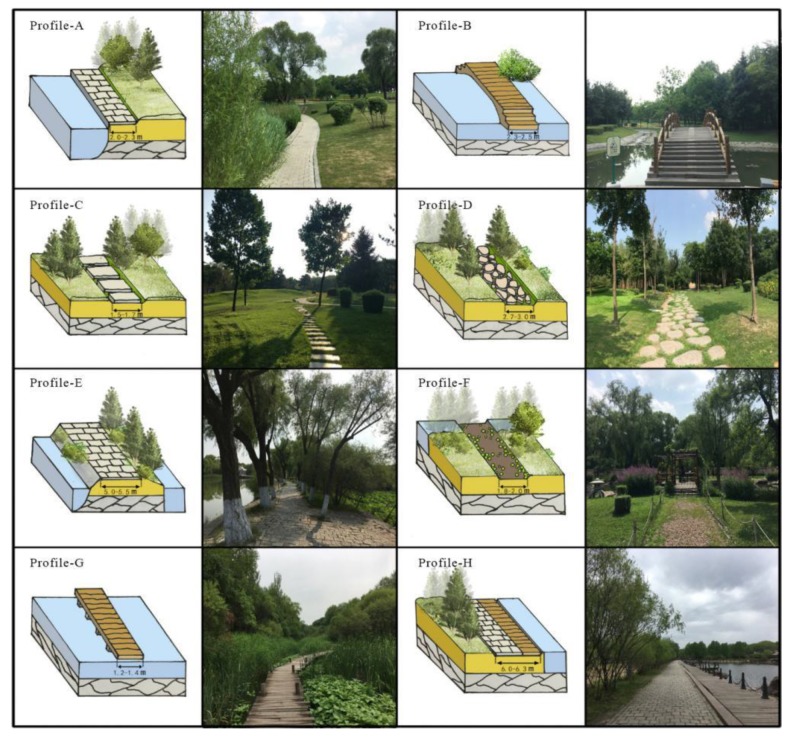
Profile pattern.

According to our field survey, garden roads divide road sections in each of the four scenic areas of Sun Island where birds are most commonly seen, with their intersections serving as boundaries and each road section defined as a unit of space. From the relationships between the common unit spaces identified using Depth map software, a convex-shaped map was generated, the values of three indicators (connection degree, integration degree, and depth) were calculated for each path, and connections were selected by the superpositions of nine paths with high degrees of connection, high levels of integration, and low depths, including three spaces in forests, wetlands, and waterfront areas. As some of these spaces were too dangerous to access (i.e., the latter path), they were omitted from the analysis, leaving a total of eight test path sections that were studied (Figure 2).

We collected images from 9:30–10:30. We collected data on sunny days but avoided doing so on cloudy and rainy days. Each image was set to the same wide angle, focused at the same height (approximately 1.5 m from the ground) and taken without a flash.

### 2.2. Sample Characteristics

**Figure 3:**
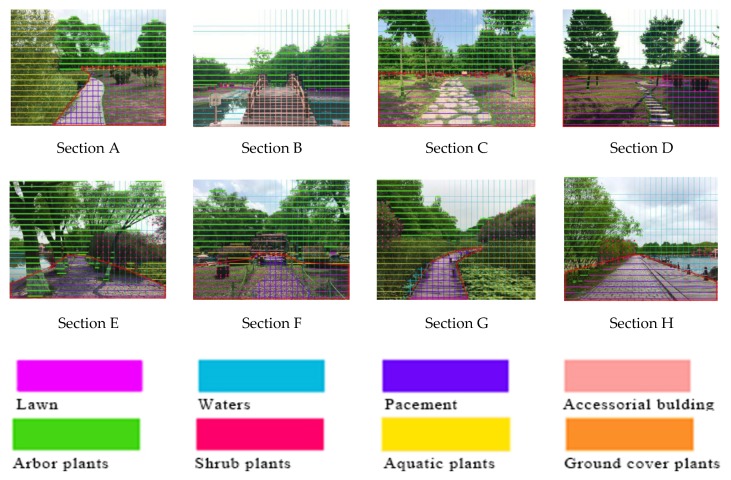
Schematic diagram of experimental path grid pixels.

The grid pixel method used by H. Nordh [31] was employed to quantify the landscape elements of the selected sections and summarize their spatial landscape characteristics. First, each section was divided into a grid of 588 (21 × 28) squares, with each square sized 5 × 5 mm^2^ and each section area covering 14,700 mm^2^. Next, different landscape elements were selected as variables (i.e., hard surfaces such as pavement and sketch; soft surfaces such as grassland, groundcover, shrubs, trees, and aquatic plants; water bodies; and sky), and different colors were used to distinguish between landscape elements. The images were then marked and the numbers of relevant pixels were calculated, as shown in Figure 3.

Table 1 presents the landscape characteristic index statistics for the sampled path section. The sky index refers to the proportion of the sky visible to the human eye from the observation point, which is generally expressed by a solid angle. When using a two-dimensional image as the evaluation medium, the perspective deformation of an image edge is disregarded and the ratio of the grid area occupied by the sky to the area of a whole image can be used to reflect the sky’s width. The formula is as follows:

(1) $$\mathrm{Sky}\;\mathrm{index}=\frac{\mathrm{Number}\;\mathrm{of}\;\mathrm{sky}\;\mathrm{pixels}}{\mathrm{Total}\;\mathrm{pixels}}*100\%.$$

In this formula, the number of sky pixels represents the grid area occupied by the sky, and the total number of pixels represents the area of the whole image.

The soft/hard ratio is the ratio of soft to hard grid areas in a two-dimensional image. The formula is written as

(2) $$\mathrm{Soft}\;\mathrm{hard}\;\mathrm{ratio}=\frac{\mathrm{Number}\;\mathrm{of}\;\mathrm{soft}\;\mathrm{pixels}}{\mathrm{Number}\;\mathrm{of}\;\mathrm{hard}\;\mathrm{pixels}}*100\%.$$

In this formula, the number of soft pixels represents soft surfaces, including trees, shrubs, herbs, and turf grid areas; and the number of hard pixels represents hard surfaces, including roads, pavement, and buildings. From these two indexes, we can measure the growth of tree and shrub layers and the distribution and proportion of elements in the path space.

The vertical coverage reflects the growth of vegetation on the site. For plants and shrubs, the line intercept method is more accurate. We estimate the vertical coverage of trees by measuring the photos. Similarly, we use the grid pixel method to calculate the vertical coverage of herbs, shrubs, and trees on the site and then calculate the total coverage of plants. Because sampling points in the sampling interval can be affected by other plants, the area occupied by other types of plants in the sampling interval is removed through percentage calculation.

### 2.3. Selection of Sound Sources

From data on the most common birds encountered on Sun Island [31], we selected four common birdsongs as our sound sources: sparrows (tree sparrow), woodpeckers (big spot woodpecker), magpies, and crows (small beaked crow). Using a high fidelity recorder (Fostex fr-2le, Akishima, Japan) to record birdsong sound frequencies at different periods, the frequencies of the four kinds of birds ranged from 1600 to 8000 hz with sound pressure levels of 50–60. These were compared with the local birdsong sound database of China (www.aigei.com), and network birdsong sound database was selected. Adobe Audition software (which can provide advanced functions including audio editing, environment mixing, controlling, and effects processing) was used to synthesize comprehensive sound variables. The parameters of research tools and instruments are shown in Table 2.

### 2.4. Experiment Flow

**Figure 4:**
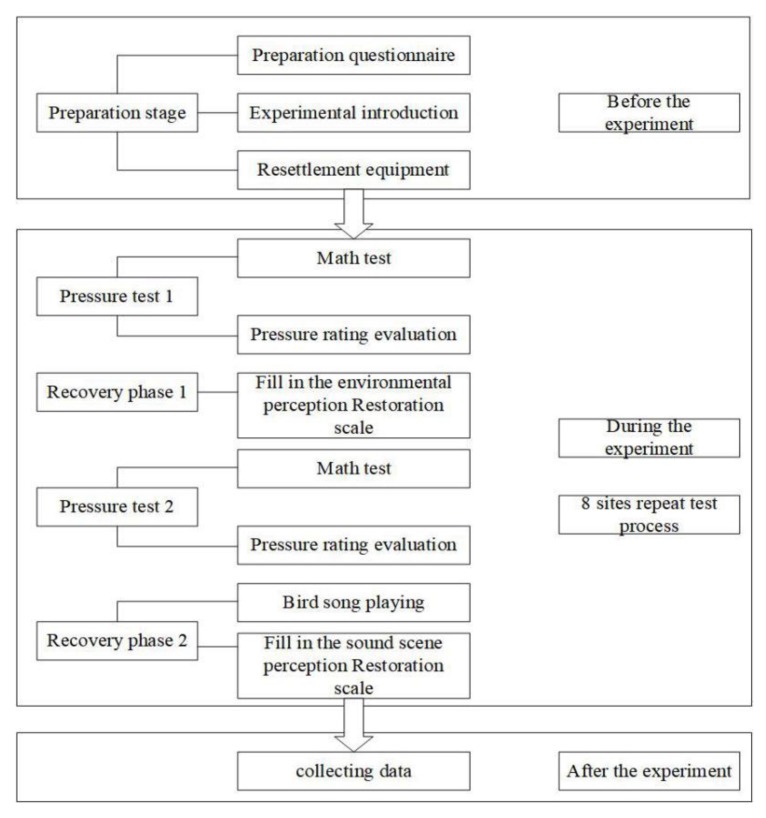
Field experiment process.

To enhance the authenticity of the data, experiments were carried out in the field. The flow is shown in Figure 4. Eight nodes in the park were selected to find tourists to answer the questionnaire. The researcher first introduced the questionnaire and had participants answer questions on their personal backgrounds and recent stressful encounters. The researcher then initiated a two-minute arithmetic problem pressure stage and asked participants to complete the (Perceived Restorativeness Scale (PRS)) environmental perception recovery semantic scale regarding their views on the surrounding environment and semantics (Table 3). This scale was developed by HARTING (1996) on the basis of four basic characteristics of restorative environments proposed by Kaplan and his wife, and was later adapted by Laumann and R. Pals et al. to form a complete restorative scale. Attractiveness represents a fascination with an environment, measuring the attraction an individual feels to the surrounding environment and its ability to stimulate pleasant positive emotions. Being away represents the stability within an environment, aiming to measure the degree of relaxation in the environment. The more relaxed a person is in an environment, the less psychological pressure and reduced fatigue they experience. Extensibility represents the spatial connection in an environment and aims to measure the scale of the environment while improving the space-time dimension of people’s imaginations and the potential for exploration. Compatibility represents tolerance in an environment, aiming to measure comfort in the surrounding environment including an individual’s sense of harmony and immersiveness [1]. Thirteen points were used in the semantic scales. The semantic scale was divided into seven levels from +3 to −3 to indicate strongly agree to strongly disagree, respectively. Finally, the two-minute arithmetic question pressure stage was completed again using a small audio device (JBL flip4 /State of California, USA) to play a recorded birdsong at a height of 4 m. After the birdsong was played, the Perceived Restorativeness Soundscape Scale included in the soundscape recovery section of the questionnaire was completed (the scale includes 13 questions with each characteristic measured with two to three questions).

### 2.5. Selection of Subjects and Stress Analysis

**Figure 5:**
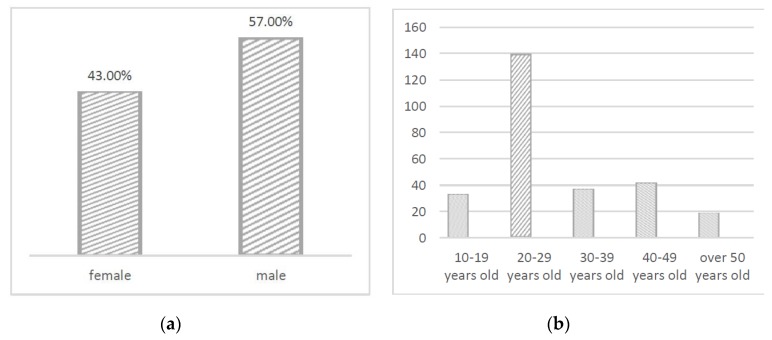
Visitor distribution. (**a**) Gender ratio; (**b**) Age ratio.

In this experiment, participants were randomly selected from eight sites on legal holidays from June to August. A total of 251 questionnaires were distributed during the field survey, 240 of which returned valid results, as shown in Figure 5. According to data statistics, 43.0% of the sample was female and 57.0% was male; participants were divided into six age groups with people aged 20–29 forming the largest cohort and accounting for 51.5%. Statistics on the frequency of park use showed that 12.4% of participants visited the park twice a month, 33.6% visited monthly, 40.5% visited every other month, and 13.5% were foreign tourists.

The validity of the Perceived Restorativeness Soundscape Scale (PRSS) and Perceived Restorativeness Scale (PRS) questionnaires were tested. The results showed a PRSS Cronbach’s α value of 0.938 with a Kom value of 0.78, and a PRS Cronbach’s α value of 0.815 with a Cronbach’s α value of 0.927. Cronbach’s α values for all of the data were higher than 0.7, satisfying reliability and validity tests and indicating that the data were accurate and effective.

The subjects’ subjective stress levels were recorded. Levels of stress affecting the participants in the last month were identified through the questionnaire, with moderate levels of stress reported by the largest number of participants, accounting for 52.9%; high levels of stress being the second most common, accounting for 23.2%; very high levels of stress being the least common, accounting for 4.0%; relatively low levels of stress representing 13.6%; and those experiencing no stress accounting for 6.3%. These data were used to better understand daily levels of stress experienced the subjects.

We heightened the subconscious stress levels of the participants by having them complete 200 mathematical arithmetic problems in two minutes. (Please refer to the attached Appendix A for details of math problem description. Question type 1: addition and subtraction; Question type 2: draw a line under the number whose addition of adjacent numbers is equal to 10. See Table A1 and Table A2 in the Appendix A.) After this stress-inducing test of math questions, 87.0% of the subjects’ stress levels were very high, relatively high, or moderate. Before the test, 80.1% of subjects rated their stress levels as high, moderate, or low. A comparison of data collected before and after the experiment showed that this method of inducing stress was very effective at increasing stress levels in most of the subjects.

## 3. Results

### 3.1. An Analysis of Perceived Recovery Benefits of Park Paths

**Figure 6:**
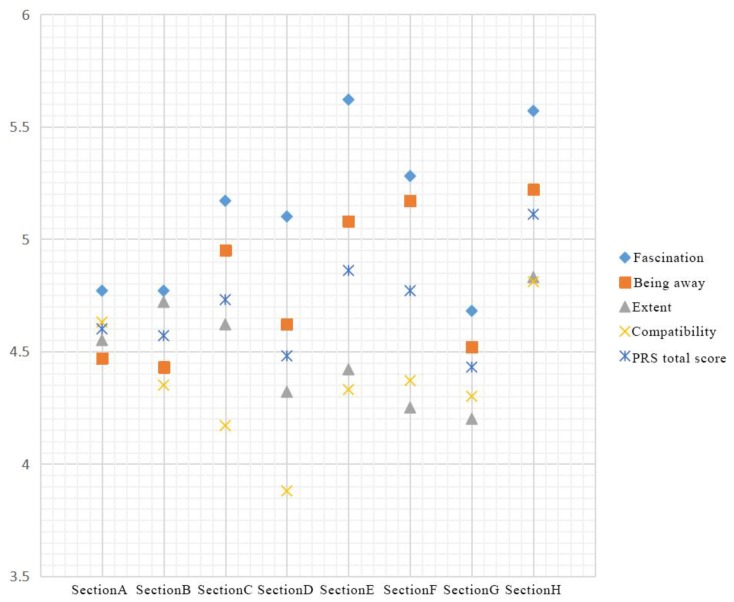
Statistical table of visual landscape perception recovery scale (PRS).

The total score for landscape perception restoration is the mean of scores measured for the eight sections. Statistical analysis of variance for the data of perceptual landscape restoration factors was conducted. The results show that there was no significant difference among different profile samples for Compatibility of 1 item in total, and there were significant differences among each profile sample for Fascination, Being away, Extent, and PRS of 4 items in total, as shown in Table A3 in the Appendix A. Figure 6 shows that section H had the highest average score of 5.11, while section G had the lowest average score of 4.43. This shows that large areas with water and tall trees can quickly calm park visitors, relieve pressure and tension, and maximize the benefits of perceived recovery.

The evaluation results of the perceived landscape restoration factors focused on the following: Fascination, Being away, Compatibility, and Extent. Kruskal–Wallis test statistics were added to the analysis as shown in Table A4 in the Appendix A. The analysis shows that there was no significant difference between two items of Compatibility and Extent in different profile samples. In addition, there were significant differences among the three sections of Fascination, Being away and PRS. The following analysis is presented:

(1) Fascination: The scores of sections E and H were relatively high, at 5.62 and 5.57, respectively. Both of these sections are positioned close to a calm lake, indicating that natural water environments enhance perceived restorative effects by increasing landscape attractiveness, adding interest, promoting interaction, and attracting visitors. Section G is located in a wetland environment and presented the lowest score of 4.68. Landscapes on both sides of the road are relatively simple, leaving levels of attraction quite low.
(2) Being away: Scores for sections F and H were high, at 5.17 and 5.22, respectively. One of these sections is located in a quiet environment with beautiful scenery, while the other is positioned near a calm lake, showing that beautiful, secluded spaces and calm water bodies encourage visitors to remain in spaces for longer periods of time. Scores for sections 1 and 2 were low, showing that the presence of paths encourages people to pass through quickly.
(3) Compatibility: The compatibility scores of each section were almost identical, showing that when the compatibility of park paths is high, the surrounding landscape conforms to the overall environment and can mobilize crowds.
(4) Extent: Section H had a score of 4.82, showing that the restorative effect of this section was relatively high. The section is positioned close to a large lake. Water bodies can enhance a sense of closeness between humans and nature, indicating that people become more immersed in such environments. Section 4 presented the lowest value of 3.88, showing that paths surrounded by treed grassland cause people to feel more detached.

### 3.2. Benefit Analysis of Perceived Restorative Effects of Birdsongs

**Figure 7:**
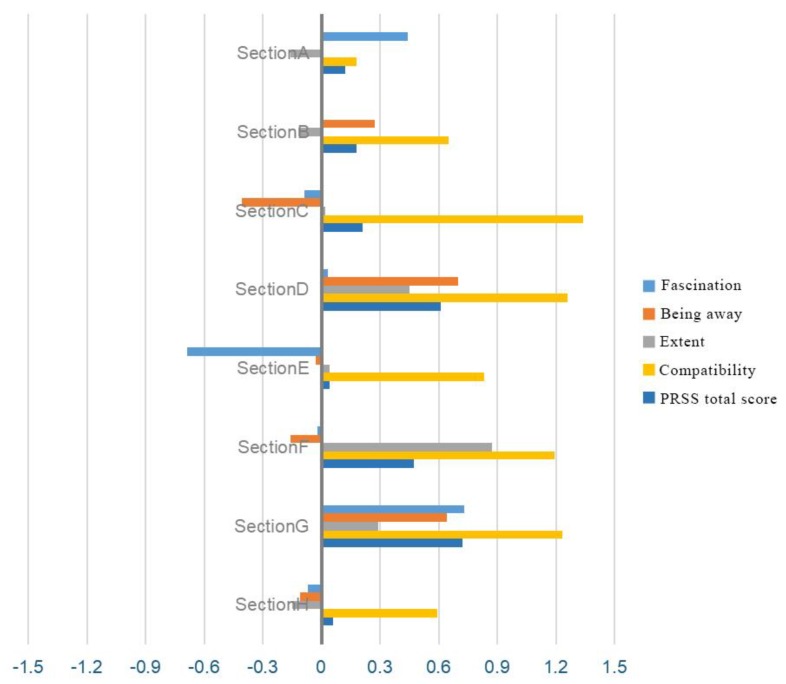
Changes in perceived resilience after introducing birdsongs (PRSS).

According to the statistical results as shown in Figure 7, the following conclusions can be drawn:

(1) Fascination: After introducing birdsongs, scores for sections A and G increased by 0.44 and 0.73, respectively, showing that the participants generally found birdsongs to create a more harmonious setting in wetland environments and that they preferred wetland landscapes with birdsongs.
(2) Being away: After introducing birdsongs, considerable increases of 0.70 and 0.64 were observed in sections D and G, respectively, showing that winding paths are more suited to bird habitat environments and allow visitors to relieve themselves of daily work stress. The score for section C was reduced by 0.41, which may have occurred because the path in this section passes through a clearing in a forest, increasing the probability of “unnecessary interference” from tourists and promoting a feeling of escape.
(3) Compatibility: After introducing birdsongs, the score for section F increased the most, by 0.87, while other scores changed less, showing that birdsongs had a limited impact on path compatibility and a certain impact on paths through dense forest, allowing visitors to quickly adjust to surrounding dense forested environments and encouraging a state of relaxation.
(4) Extent: After introducing birdsongs, extent scores changed considerably, increasing by 0.925 on average in the eight sections. The score for section C changed the most, increasing by 1.34, showing that birdsongs have a strong impact on extent values. Birdsongs can broaden a scene, increase the perceived openness of a site, and highlight the naturally artistic features of birdsongs.
(5) The total PRS score for section H was highest when birdsongs were not introduced. After introducing birdsongs, the total score for sections D and G changed the most, increasing by 0.61 and 0.72 and showing that the restorative effects of birdsongs are stronger when introducing birdsongs to path environments that include water bodies.

### 3.3. An Analysis of the Restorative Benefits of Path Spaces

**Figure 8:**
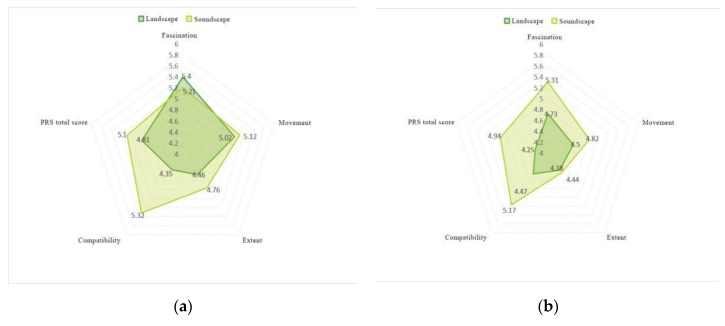

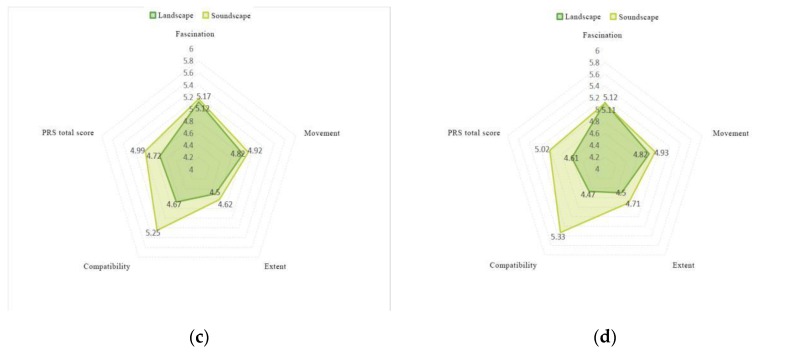
Restorative benefits observed for different paths: (**a**) water’s edge (waterfront space), (**b**) water’s edge (wetland), (**c**) waterfront space and wetland, and (**d**) clearing in the woods.

As shown in Figure 8, the eight studied sections can be divided into three types of path spaces: wetland spaces, waterfront spaces (including sections A, B, E, F, G, and H) and open spaces in forests (sections C and D). Perceived restorative effects observed before and after introducing birdsongs are shown in Figure 3. We found effects of the three types of path space on ductility to increase significantly after introducing birdsongs. Kruskal–Wallis testing was performed for each section and four characteristics, as shown in Appendix A Table A5. Of the paths through wetland and waterfront spaces and those through open forested space, scores for the paths through open forested space increased the most after introducing birdsongs. Paths through the waterfront space generated a higher overall visual perception recovery efficiency value than wetland paths. We found no significant difference between the two after introducing birdsongs, but improvements in wetland path perceptions and restorative effects were stronger than those for the waterfront space.

### 3.4. Correlation Analysis of Landscape Elements and Perceptual Restoration

In this study, correlations between PRS and PRSS data, sky indexes, soft/hard ratios, and vertical coverage were analyzed. The results show that PRS and PRSS evaluations had a significantly negative correlation with sky indexes and a significantly positive correlation with soft/hard ratios and vertical coverage. Through comparisons we found that the correlation for the perceived restorative effects of soundscapes was stronger than that for landscape perception recovery, showing that the influence of soft/hard ratios and vertical coverage on the perceived restorative effects of soundscapes was greater than the effect on landscapes. The results of the Spearman correlation analysis are shown in Table 4.

(1) There was a significant negative correlation between the sky index and each feature. The effects of the restorative characteristics of landscape perceptions are ranked as follows: Fascination > Compatibility > Being away > Extent. The effects of restorative characteristics of soundscape perceptions are ranked as follows: Fascination > Being away > Extent > Compatibility. This may be the case because park users usually remain in shaded areas. A small sky index means that the broader one’s view of a landscape, the less private that landscape is and the lower the level of psychological safety, resulting in a reduced restorative effect.
(2) The soft/hard ratio had a significant positive correlation with each property. The effects of restorative characteristics of landscape perception are ranked as follows: Fascination > Compatibility > Extent > Being away. The effects of restorative characteristics of soundscape perception are ranked as follows: Extent > Fascination > Compatibility > Being away. Relative to the soft/hard ratio, the higher the proportion is, the larger the proportion of vegetated soft and hard surfaces are in a site, the more places there are for birds to inhabit, and the higher the diversity and recovery of birdsong species.
(3) Vertical coverage had a positive correlation with each characteristic. The significance of perceived restorative landscape effects can be ranked as follows: Compatibility > Extent > Fascination > Being away. The significance of soundscape perception recovery can be ranked as follows: Fascination > Being away > Compatibility > Extent.

Vertical coverage estimates the projection ratio of the aboveground section of the vertical direction from photos. In an image, vertical coverage also indirectly affects the sky index and soft/hard ratio. Therefore, vertical coverage is the most important and influential of the three green space indexes. Vertical coverage is divided into vegetated areas and areas with shrubs and trees. The correlation between these three indexes and perception is also compared. We found the correlation between the vertical coverage of trees to be the largest, while for shrubs it was the smallest. This shows that increasing the tree layer is more conducive to perception recovery in landscape spaces, but that while the shrub layer also has effects on recovery, the presence of too many shrubs can easily block views, compromising security levels.

## 4. Discussion

### 4.1. Comparison of Perceived Visual and Auditory Restorative Benefits of Landscape Paths

The results of our comparisons of perceived recovery characteristics are as follows:

(1) Park paths have restorative benefits, and paths with tall trees and water bodies promote perceived restorative effects.
(2) Birdsongs offer a significant recovery benefit; after introducing birdsongs, perceived recovery benefits are significantly improved.
(3) When improving recovery efficiency, ductility levels are the highest, while attractiveness levels are the lowest.

### 4.2. Recovery Benefits of Birdsongs in Relation to Path Types

From comparisons of the three kinds of paths studied, the following conclusions are drawn:

(1) In terms of the restorative benefits of road space types, paths through wetlands and waterfront spaces have more benefits than paths through open spaces in forests in terms of four characteristics(Extent, Fascination, Compatibility, and Being away), but after introducing birdsongs, three characteristics (escape, compatibility, and extension) are greatly improved relative to the original values, though the difference found between the two types of paths is minor.
(2) The effects of path types on restoration efficiency differ. Wetland and waterfront spaces present higher path values than open spaces in forests, and restorative effects are positive. Birdsongs have a stronger impact on the perceived restorative effects of site, but the effect on path types is not significant.
(3) Under original landscape conditions, paths through waterfront spaces have more restorative effects than paths through wetlands; after introducing birdsongs, wetland paths have more restorative effects than waterfront paths.

### 4.3. Landscape Characteristic Indexes and Restorative Benefits

From the above correlations, the following inferences can be made:

(1) The lower the sky index, the higher the proportion of soft and hard surfaces, the greater the degree of vertical coverage, and the more obvious the perceived restorative effect.
(2) The effect of the soft/hard ratio and vertical coverage on the restoration of visual interactive perceptions is stronger than that of visual landscape perceptions. Total PR and PRS scores are higher, and the restorative effect is stronger.
(3) The proportion range is 29.9–65.6%. The higher the proportion of trees and shrubs present, the stronger the benefits of perceptual restoration.

### 4.4. Path Space Patterns of the Perceived Restorative Effects of Birdsong Soundscapes

**Figure 9:**
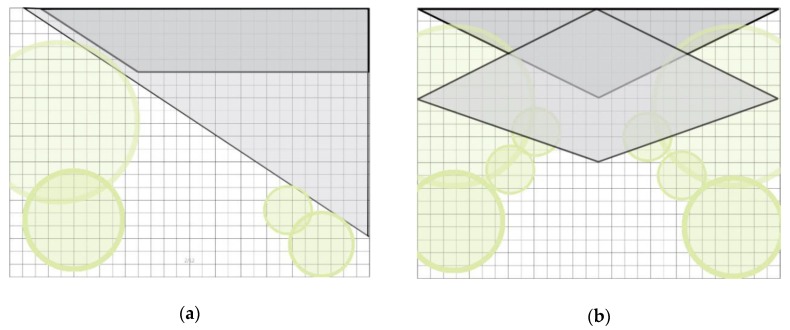
Different space types: (**a**) environmental mode one side; (**b**) environmental mode bilateral.

**Figure 10:**
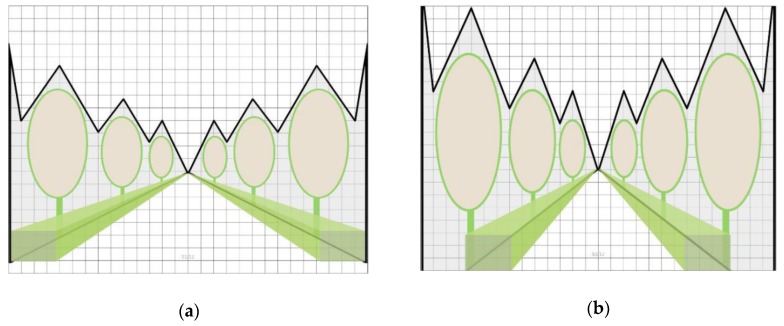
Taking the simplest planting method as an example: (**a**) vegetation pattern minimum; (**b**) vegetation pattern maximum.

**Figure 11:**
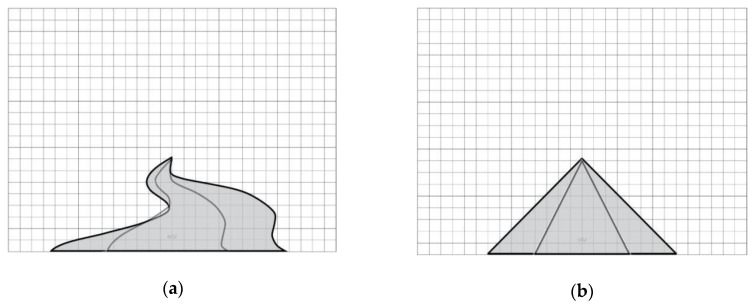
Taking the simplest path mode as an example: (**a**) path mode curve; (**b**) path mode line.

(1) Environmental model: When the sky index range is between 13% and 36%, the recovery effect is stronger. Therefore, an environmental model maximizing the recovery benefit is proposed. Gray areas in two kinds of environment models represent the sky area, which are divided into one side and two sides, with different lines expressing the value ranges of the sky. The proposed environmental models based on different space types are shown in Figure 9.
(2) In the vegetation model, when the vertical coverage range is 30.28–65.65%, the recovery effect is stronger; when the vertical coverage of trees is 30.28–38.6% and the vertical coverage of trees and shrubs is 32.5–65.6%, the recovery effect is stronger. Based on these data, the following two vegetation models (taking the simplest planting method as an example) are proposed to maximize the restorative effects of shrubs and trees, as shown in Figure 10.
(3) In the path mode, when the soft/hard ratio ranges from 5–21, the recovery effect is strong. Therefore, according to scale data used to calculate the path size according to the range requirements, two kinds of path modes are proposed to maximize the recovery benefit (taking the simplest path mode as an example), as shown in Figure 11.

## 5. Conclusions

Based on past research, this study involved a comparative analysis of the perceived restorative benefits of birdsongs encountered in urban parks and a correlation analysis of quantitative path space morphological characteristics and their perceived restorative effects. The following conclusions are drawn:

(1) Among the perceived restorative effects observed through audio-visual interactions, birdsongs significantly improved perceived restorative effects, and birdsongs had the strongest impact on ductility.
(2) After introducing birdsongs, the restorative effects of wetland path spaces were the strongest; for different types of path restorative effects, the difference was not significant.
(3) The proportion of soft and hard surfaces present was positively correlated with the perceived recovery benefit. When the value was no less than 5, the perceived restorative effect was enhanced. When the proportion of trees and shrubs was 29.9–65.6%, the perceived restorative benefit was the strongest, showing that planting trees and shrubs along a path is the most desirable within this range. The sky index was negatively correlated with the perceived recovery benefit, and the perceived recovery benefit was most significant when the value was 13–36. This shows that the overall crown threshold for the planting of tall trees along a path should not be too high, and that the proportion of sky should be between 42% and 68%.

In this study, we did not apply detailed classifications of birdsongs. In the future, we will conduct further quantitative analyses of sound sources. In addition, as physiological instruments and index data fluctuated considerably in our field test, we used a questionnaire to measure perceived recovery validity. Future studies may explore the impacts of different types of birdsongs encountered in urban parks on people’s perceptions of recovery to better design restorative environments.

## Figures and Tables

**Table 1:** Landscape characteristic index statistics of the sample section (percentage).

Profile	Path Type	Prime Number of Trees and Shrubs	Number of Path Pixels	Soft/Hard Ratio	Vertical Coverage (%)	Sky Index (%)
Section A	Waterfront wetland (open)	135	53	8.55	37.62	13.95
Section B	Waterfront trestle	159	83	3.01	28.19	43.37
Section C	Treed grassland	186	78	14.83	56.31	24.66
Section D	Treed grassland	167	24	14.31	30.28	15.14
Section E	Waterfront trail	386	118	3.60	61.10	6.12
Section F	Waterfront	247	56	5.41	61.29	13.95
Section G	Wetland	178	60	6.68	65.65	21.60
Section H	Waterfront (open)	146	133	1.57	26.67	36.73

**Table 2:** List of experimental instruments.

Instrument	Model and Origin	Purpose	Parameter Range
**Sound meters**	BSWA801/China	Measurement of sound pressure level and frequency	Measure: 24 dBA~140 dBA; Frequency: 0.5 Hz~20 kHz
**High fidelity recorder**	FOSTEX FR-2LE/Japan	Record birdsong	Recording frequency: 20 Hz–20 kHz ± 2 dB (FS 44.1/48 kHz)
**3D camera**	Fuji 3DW1/China	3D photo shooting	Effective pixels: 10 megapixel; Highest resolution: 3648 × 2736
**Mini audio**	JBL Flip4/America	Playing processed audio	Frequency response: 70 Hz–20 KHz

**Table 3:** Semantic descriptions of environmental restoration.

Restorative Environmental Characteristics	Restorative Experience with the Landscape	Semantic Description
Fascination	The appeal of the environment	Like - dislike Beautiful - mediocre
Being away	A sense of relaxation	Stay - leave Comfortable - uncomfortable
Compatibility	Exploration of features	Rich - monotonous Fun - boring
Extent	An intoxicating environment	Harmony - conflict Immersion - separation

**Table 4:** Landscape and sound perception restoration.

Variable Name		Fascination	Being Away	Extent	Compatibility	PRS/PRSS
Sky index	correlation coefficient (landscape)	−0.564 **	−0.452 **	−0.443 **	−0.512 **	−0.611 **
	correlation coefficient (soundscape)	−0.660 **	−0.535 **	−0.412 **	−0.389 **	−0.739 **
Soft/hard ratio	correlation coefficient (landscape)	0.721 **	0.343 **	0.385 **	0.574 **	0.539 **
	correlation coefficient (soundscape)	0.577 **	0.433 **	0.628 **	0.541 **	0.668 **
Vertical coverage	correlation coefficient (landscape)	0.565 **	0.475 **	0.587 **	0.621 **	0.613 **
	correlation coefficient (soundscape)	0.711 **	0.643 **	0.457 **	0.627 **	0.704 **
Tree vertical coverage	correlation coefficient (landscape)	0.621 **	0.443 **	0.475 **	0.642 **	0.679 **
	correlation coefficient (soundscape)	0.644 **	0.463 **	0.396 **	0.484 **	0.539 **
Shrub vertical coverage	correlation coefficient (landscape)	0.501 **	0.383 **	0.512 **	0.597 **	0.522 **
	correlation coefficient (soundscape)	0.621 **	0.644 **	0.458 **	0.534 **	0.539 **
Herbaceous vertical coverage	correlation coefficient (landscape)	0.533 **	0.463 **	0.379 **	0.572 **	0.596 **
	correlation coefficient (soundscape)	0.618 **	0.435 **	0.523 **	0.412 **	0.539 **

Note: ** When the confidence value (double test) is 0.01, the correlation is significant.

## Appendix A

**Table A1:** Answer the following 100 arithmetic questions in 2 min.

12 + 68 =	22 + 70 − 50 =	67 − 34 =	61 − 10 =	80 − 72 + 18 =	83 − 24 =
78 − 23 =	88 − 43 − 31 =	76 − 26 =	59 + 6 =	70 − 48 =	56 + 12 =
12 + 48 =	34 + 40 − 13 =	84 − 66 =	66 − 33 =	79 − 40 =	99 − 9 =
25 − 14 =	75 − 51 + 52 =	39 + 8 =	51 + 5 + 35 =	72 − 54 =	35 − 28 =
27 + 56 =	6 + 59 =	73 − 41 =	50 − 5 − 15 =	33 + 38 =	32 − 29 =
54 − 45 =	58 − 13 =	68 − 40 =	62 − 29 − 29 =	81 − 36 =	63 + 10 =
13 + 49 =	14 + 53 =	16 + 27 =	54 − 32 + 45 =	92 − 38 =	97 − 35 =
65 − 27 =	89 − 9 =	89 − 9 =	98 − 97 + 39 =	60 − 9 =	97 − 49 =
14 + 35 − 28 =	5 + 83 =	73 − 21 =	96 − 43 =	62 − 33 =	13 + 19 − 4 =
23 + 32 =	86 − 7 =	93 − 52 =	80 − 18 =	39 − 15 =	27 + 47 − 23 =
55 + 36 =	62 − 27 =	28 − 8 =	45 − 41 =	41 − 5 =	12 + 9 − 6 =
29 + 37 =	17 − 13 + 64 =	19 − 12 =	16 + 8 =	43 − 29 =	57 − 16 − 7 =
28 + 64 =	85 − 39 + 42 =	92 − 36 =	84 − 73 =	53 − 5 =	67 − 12 =
41 + 38 =	78 − 25 + 15 =	30 + 44 =	25 + 31 =	25 + 44 =	29 + 34 =
69 − 58 =	19 + 27 − 41 =	39 + 35 =	99 − 75 =	86 − 70 =	43 − 7 =
58 − 28 =	23 + 36 − 44 =	50 − 9 =	20 + 16 + 13 =	74 − 8 =	93 − 61 =
33 − 24 =	15 + 67 − 34 =	69 − 7 =	46 + 39 − 53 =	47 + 29 =	17 + 8 + 4 =

**Table A2:** In the following 15 lines of numbers, there are 80 groups of adjacent numbers that add up to 10. Underline each group of numbers that meet the requirements, such as 2357 **64** 389 **82** 26 **91**.

A .8 9 2 4 6 7 3 6 3 2 4 6 2 8 8 2 1 2 6 4 5 6 7 8 9 8 7 6 5 4 3 7
B .3 7 7 6 5 5 3 2 1 9 1 7 6 5 3 3 3 7 3 1 4 2 1 2 2 1 8 2 9 1 0 3
C .2 5 6 7 8 9 0 3 4 5 4 8 2 7 3 5 9 3 0 2 7 3 5 4 9 8 2 7 3 8 4 6
D .6 4 8 2 4 6 8 0 9 1 3 3 5 5 9 1 0 2 3 8 2 8 7 3 0 9 7 5 7 4 9 1
E .6 8 5 4 8 2 6 5 1 4 5 2 1 5 2 2 1 6 8 7 9 1 8 2 7 6 5 5 9 8 4 2
F .5 8 4 5 6 4 5 5 4 5 7 8 2 9 1 5 4 2 6 2 2 3 5 1 2 5 5 7 3 9 1 8
G .9 1 3 7 8 0 2 1 4 6 6 5 2 5 4 1 6 4 2 8 9 4 5 6 5 5 2 7 9 5 6 8
H .8 5 1 2 6 7 3 9 0 1 9 8 7 2 4 6 3 7 5 4 5 5 8 7 2 1 9 8 2 5 6 7
I .1 1 5 7 2 6 4 7 3 8 2 9 1 4 6 8 8 7 6 5 4 9 3 4 7 2 5 5 6 4 7 3
J .4 5 9 7 2 5 5 6 4 6 5 2 1 2 6 4 1 9 2 8 6 4 5 5 7 3 1 0 2 5 4 8
K .4 7 8 5 4 6 9 1 2 8 5 6 7 3 0 4 0 9 7 2 6 4 8 0 2 9 1 0 4 6 5 3
L .5 8 2 4 6 9 0 5 0 7 0 8 0 6 0 4 0 9 1 7 3 4 6 9 1 5 9 7 5 4 5 8
M .5 7 3 4 9 7 6 8 4 1 7 3 8 2 4 9 7 5 4 6 5 5 4 6 7 6 5 4 5 5 7 9
N .9 1 8 4 6 4 5 7 3 2 0 6 7 5 4 9 1 2 8 7 5 4 6 0 1 8 2 7 3 6 4 5
O .4 8 4 6 1 9 7 5 9 8 1 9 4 7 8 9 6 4 5 5 8 4 3 2 8 7 3 6 4 1 8 6

**Table A3:** ANOVA results.

	F	*p*
Fascination	4.533	0.000 **
Being away	3.136	0.003 **
Extent	1.494	0.17
Compatibility	2.164	0.038 *
PRS	3.095	0.004 **

* *p* < 0.05 ** *p* < 0.01.

**Table A4:** PRS Kruskal–Wallis results.

Kruskal-Wallis Results										
	Section								Kruskal-Wallis Test Statistics	*p*
	A (*n* = 30)	B (*n* = 30)	C (*n* = 30)	D (*n* = 30)	E (*n* = 30)	F (*n* = 30)	G (*n* = 30)	H (*n* = 30)		
Fascination	4.750	5.000	5.500	5.500	5.500	5.500	4.750	6.000	31.929	0.000 **
Being away	4.500	4.500	5.250	4.500	5.500	5.000	4.500	5.500	22.581	0.002 **
Extent	4.500	4.750	4.500	4.250	4.500	4.500	4.500	4.750	8.781	0.269
Compatibility	4.500	4.500	4.500	3.500	4.500	4.250	4.500	4.500	13.510	0.061
PRS	4.620	4.750	4.815	4.560	4.880	4.815	4.500	5.060	18.898	0.009 **

** *p* < 0.01.

**Table A5:** PRSS Kruskal–Wallis results.

Kruskal–Wallis Results										
	Section								Kruskal-Wallis Test Statistics	*p*
	A (*n* = 30)	B (*n* = 30)	C (*n* = 30)	D (*n* = 30)	E (*n* = 30)	F (*n* = 30)	G (*n* = 30)	H (*n* = 30)		
Fascination	5.330	5.000	5.000	5.330	5.165	5.330	5.670	6.000	10.725	0.151
Being away	4.500	5.000	4.375	5.500	5.125	5.125	5.125	5.625	20.197	0.005 **
Extent	4.500	4.670	4.835	4.670	4.670	5.330	4.670	4.670	10.710	0.152
Compatibility	4.835	5.000	5.835	5.330	5.330	5.835	5.500	5.500	15.917	0.026 *
PRS	4.540	4.890	5.020	5.250	5.115	5.390	5.250	5.325	11.539	0.117

* *p* < 0.05 ** *p* < 0.01.
